# Instantaneous 4D micro-particle image velocimetry (µPIV) via multifocal microscopy (MUM)

**DOI:** 10.1038/s41598-022-22701-3

**Published:** 2022-11-02

**Authors:** M. G. R. Guastamacchia, R. Xue, K. Madi, W. T. E. Pitkeathly, P. D. Lee, S. E. D. Webb, S. H. Cartmell, P. A. Dalgarno

**Affiliations:** 1 EPSRC Centre for Doctoral Training in Applied Photonics, Heriot-Watt University, Edinburgh, UK; 2 Science and Technology Facilities Council, Research Complex at Harwell, Rutherford Appleton Laboratory, Harwell, UK; 3 Department of Materials, School of Natural Sciences, Faculty of Science and Engineering, University of Manchester, Manchester, UK; 4 The Henry Royce Institute, Royce Hub Building, The University of Manchester, Manchester, UK; 5 Institute of Biological Chemistry, Biophysics and Bioengineering, Heriot-Watt University, Edinburgh, UK; 6 Present Address: 3Dmagination Ltd, Atlas Building, Harwell Campus, Didcot, UK; 7 Present Address: Biotechnology and Biological Sciences Research Council, Swindon, UK

**Keywords:** Fluorescence imaging, Microscopy, Biomedical engineering

## Abstract

Multifocal microscopy (MUM), a technique to capture multiple fields of view (FOVs) from distinct axial planes simultaneously and on one camera, was used to perform micro-particle image velocimetry (µPIV) to reconstruct velocity and shear stress fields imposed by a liquid flowing around a cell. A diffraction based multifocal relay was used to capture images from three different planes with 630 nm axial spacing from which the axial positions of the flow-tracing particles were calculated using the image sharpness metric. It was shown that MUM can achieve an accuracy on the calculated velocity of around (0.52 ± 0.19) µm/s. Using fixed cells, MUM imaged the flow perturbations at sub-cellular level, which showed characteristics similar to those observed in the literature. Using live cells as an exemplar, MUM observed the effect of changing cell morphology on the local flow during perfusion. Compared to standard confocal laser scanning microscope, MUM offers a clear advantage in acquisition speed for µPIV (over 300 times faster). This is an important characteristic for rapidly evolving biological systems where there is the necessity to monitor in real time entire volumes to correlate the sample responses to the external forces.

## Introduction

As a potent mechanical stimulus to bone tissue, fluid-induced shear stress (FSS) has been shown to trigger a wide variety of cellular behaviour changes in vitro^1^. To study the process of translating mechanical stimulation to cellular response (i.e. mechanotransduction), parallel plate flow chambers (PPFC) have been widely employed to apply mechanical stimuli to cells via the perfusion of culture media^2,3^. Due to its simple geometry, the flow velocity and the FSS on the cell-seeded channel wall (i.e. wall shear stress, WSS) inside a PPFC are often analytically estimated in literature. However, this approach is known to overlook the perturbation to the flow caused by the cells in the PPFC^4,5^.

An imaging technique called micro-particle image velocimetry (µPIV^2,6^) is able to characterise the flow at microscale level, thus, it can be used to investigate and monitor the behaviour of the local flow around the cells in mechanotransduction studies. In µPIV, the local flow field can be reconstructed through tracking in real time the movement of micro-sized beads in a 3D volume of space via a microscope. Commonly, this is achieved through either continuously z-scanning the imaging plane axially or by imaging the flow at different axial positions over fixed intervals of time^4,7–9^. In both cases, however, it is not possible to image the entire volume simultaneously since the data on each imaged plane cannot be acquired at the same time. This, in turn, could lead to the loss of information (ambiguous spatio-temporal localisations) in the case of evolving biological systems that require fast (high temporal resolution) and continuous monitoring of entire 3D volumes. In addition, the time required to move and stabilise the objective lens across the different positions is also limiting. To partially overcome these limitations, point spread function (PSF) engineering techniques that, for example, exploit astigmatic or double-helix PSFs could be used^10,11^, since they probe volumetric space in real time. However, although highly accurate, these methods are typically limited to axial depths around 1–2 μm, thus precluding the observation of some phenomena (e.g. intercellular molecule transfer^12^) that can occur over longer axial ranges.

Multifocal microscopy (MUM) is a technique that enables multiple object planes to be simultaneously observed on a single camera. Diffractive optical element (DOE) approaches provide a simple yet effective method compatible with standard widefield microscope platforms^13–15^. In this work, we show that by combining MUM, µPIV and axial localisation techniques, an axially extended 3D volume can be observed simultaneously with high acquisition speed required for many biological applications. A multifocal system of M planes is nominally M times faster than a single-plane widefield one, assuming preservation of photon count in each imaged plane. In practice, though, photon flux is split over the M planes, thus reducing SNR, and we show how this needs to be considered alongside the speed gain due to the increasing the number of imaged planes.

This paper describes the application of multifocal microscopy (MUM) as a potential method to perform µPIV to reconstruct velocity and shear stress maps around two different case study cell samples. MUM was firstly validated as a microscopy method for µPIV against the expected theoretical behaviour and that observed with the current standard—confocal laser scanning microscope. Then, it was used on a fixed HeLa cell to reconstruct velocity and shear stress fields at the cell edge. Finally, it was showcased with live murine osteoblasts MC3T3-E1 and human mesenchymal stem cells (hMSC) during perfusion in vitro. We demonstrate that MUM based µPIV has a promising axial accuracy of over 300 times faster acquisition speed compared to confocal laser scanning microscope.

## Methods

### Experimental apparatus

**Figure 1:**
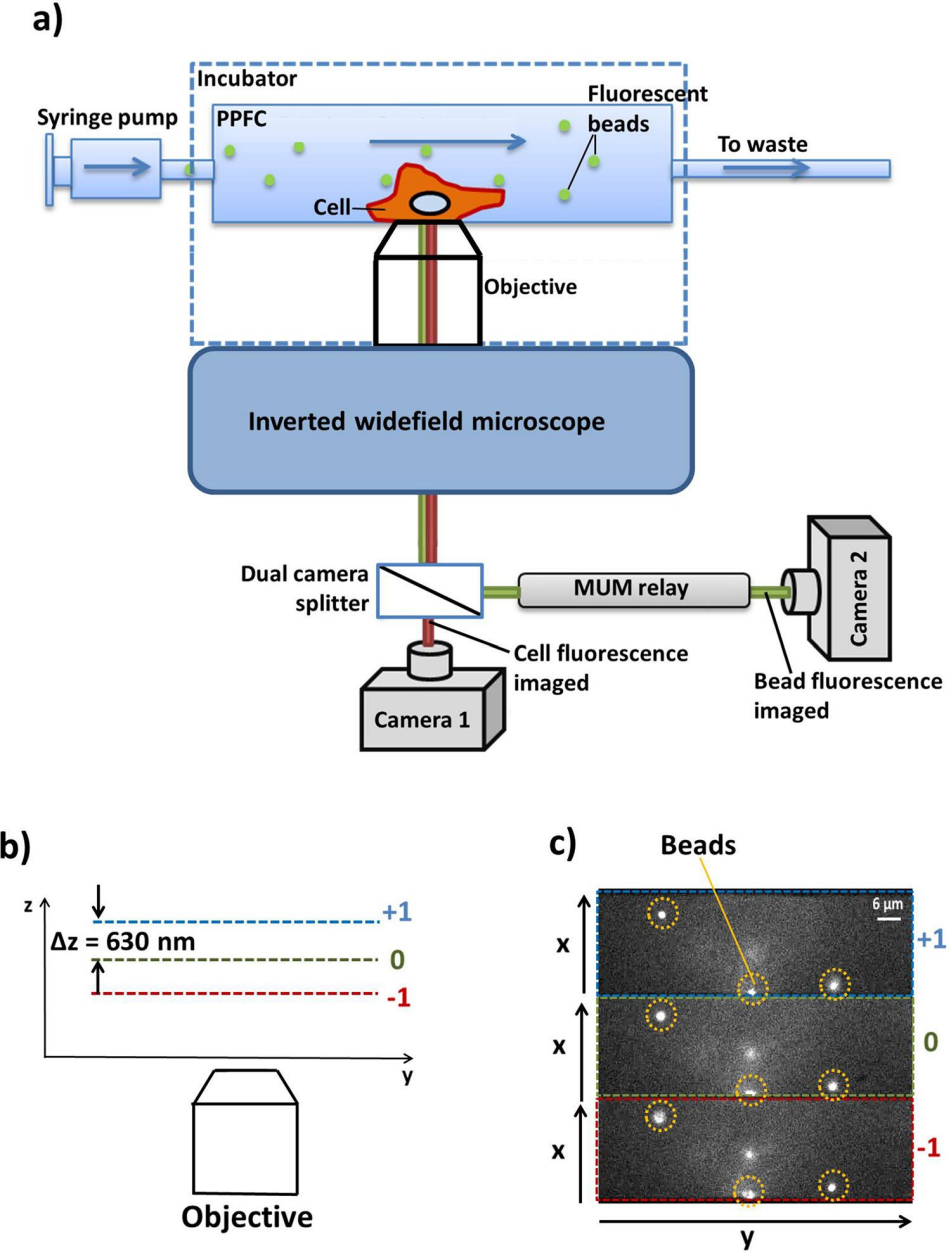
(**a**) Schematic representation of the MUM setup used to perform µPIV. The dual camera splitter and Camera 1 were added only for the live cell experiment. (**b**) Not-to-scale schematic of the axial position of the three planes acquired simultaneously with the MUM setup. (**c**) Example camera image showing beads acquired with the MUM setup. The three different planes imaged simultaneously are indicated.

The experimental system was composed of a commercial widefield Zeiss Axio Observer microscope (Axio Observer.Z1) with a 100 × 1.46 NA oil immersion objective lens (Zeiss, 440782-9800-000, diffraction limited resolution: 217 nm lateral, 740 nm axial^16^), an emCCD camera (Andor, Ixon Ultra 897) with 100 ms exposure time (10 fps) and 2000 electron multiplying (em) gain (512 × 512 pixels, pixel size = 16 μm). The MUM system consists of a telecentric^17^ diffraction based multifocal relay, which follows the exact design approach presented previously by Dalgarno et al.^13^ and presents characteristics of ease of alignment and need for a single imaging camera. In the multifocal relay (Fig. 1a), a quadratically distorted diffraction grating^18^ (DDG) is paired with a 4f optical relay to introduce a sign dependent focal power in the non-zeroth orders, which allows for the imaging of three planes, with 630 nm sample spacing simultaneously on a single camera (Fig. 1b,c). The plane spacing and relay length was chosen to fulfil the requirement to maximise the light collection of the high NA objective lens with the available grating set^13^. . Together this produced an effective axial range of 8 μm^19,20^ (fig. s1c), thanks to the optimal overlap of sharpness curves^14^ (s1a), to maximised their relative rate of change between neighbouring planes. The 4f 1:1 relay had an effective focal length of 77.87 mm, obtained by using two back-to-back achromatic doublets with focal length 150 mm (Thorlabs, AC254-150-A). A 10 nm wide 520 nm bandpass filter (Semrock, FF01-520/5-25) was used to narrow down the emission spectrum and minimise the chromatic aberration due to the DDG. For the live cell experiment, an additional detector was needed to capture the cell morphology change as well as the movement of the polystyrene beads; thus, a dual camera splitter (Cairn) was added before the multifocal relay and the emCCD camera was replaced with two digital sCMOS cameras (Hamamatsu, ORCA-Flash4.0) with 100 ms (10 fps) exposure time (2048 × 2048 pixels, pixel size = 6.5 μm). For the validation experiment, a confocal microscope (Leica, TCS SP8) equipped with a 100 × 1.40 NA oil immersion objective lens (resolution: 165 nm lateral, 610 nm axial^21^) and a hybrid detector with 100 gain at 500–550 nm wavelength range was used.

In all experiments, a syringe pump (Harvard Apparatus, 11 plus 70-2012) was used to perfuse the medium through a PPFC to an Eppendorf tube, which served as an outflow collector (Fig. 1a). PPFC with channel height of 200 µm, width of 5 mm and length of 50 mm was used (Ibidi, μ-Slide I^0.2^ Luer) for the validation experiment and the live cell experiment, while one with reduced channel height (100 µm, Ibidi, μ-Slide I^0.1^ Luer) was used for the fixed cell experiment. The perfusion medium was composed of Dulbecco’s phosphate—buffered saline (DPBS) supplemented with 250 µl of 1 µm fluorescent polystyrene beads (Thermofisher Scientific, F8823) for flow tracing and 0.3 g/ml dextran (Sigma-Aldrich, 31392-50G) to increase medium viscosity. For the live cell experiment, DPBS was replaced with Dulbecco’s Modified Eagle’s Medium (DMEM), 2% foetal bovine serum, 1% Antibiotic Antimycotic Solution and 25 mM HEPES were also supplemented to maintain cell viability. A flow rate (V_f_) of 1 µl/min was used in the validation and fixed cell experiment whilst a V_f_ of 4 µl/min was used in the live cell experiment. The WSS for the fixed cell and live cell experiment was calculated to be 1.00 and 0.86 Pa, respectively (eq. s3). The fluorescent polystyrene beads were excited with a 488 nm laser—PhoxX 488-60 (Omicron) on the MUM system and with a SuperK Evo (NKT) supercontinuum laser source on the confocal microscope.

### Flow data acquisition

For the MUM system, the field of view per plane at sample level was 81.9 × 27.3 µm^2^ in the validation and fixed cell experiment, and 129 × 26 µm^2^ in the live cell experiment. The axial range of 8 µm (fig. s1c) was used to image the axial positions ranging from 6 to 14 µm; this is because the cell top for the fixed and live cell experiment was identified at around 10 µm from the coverslip of the PPFC, where the central MUM plane was positioned. The flow was imaged for around 2 min, 30 min and 10 min respectively in the validation, fixed cell and live cell experiments.

For the flow imaged via the confocal microscope, the image size was fixed to 25.81 × 51.67 µm^2^ with 512 × 1024 pixels per acquired image (i.e., ~ 50.43 nm/pixel at sample level), in order to have a FOV comparable to that used with MUM. The acquisition speed was set to 2 kHz, the maximum, thus allowing each frame to be acquired in 261 ms. To acquire images of the bead flows with the confocal microscope over a 3D volume, the imaging plane was moved by steps of 1 µm, from the axial position 6 µm to the axial position 14 µm, thus acquiring 9 planes. Each plane was imaged for 2 min. All acquired planes were collated to create the final 3D image stack.

### Particle localisation and tracking

For the MUM experiments, the axial positions of the beads (around 20,000 localisations) in the acquired flows were calculated via the sharpness algorithm previously presented by Dalgarno et al.^14^. This algorithm relies on the calculation of the sharpness of an image^22^ as a function of its axial position through a set of sharpness calibration curves generated from reference samples. Agarose gel with refractive index (n = 1.33) close to that of the perfusion medium (n = 1.37, eq. s2) was used to create the sharpness calibration curves. The lateral positions of the beads were obtained through centre of mass (CoM) calculations, which were performed on the z-stacks resulting from the sub-pixel precise lateral registration of the images in the three acquired planes (fig. s1). The sharpness and the CoM calculations described were performed by using a custom ImageJ plugin (fig. s2a).

For the confocal microscopy system, the beads acquired were laterally localised (around 5700 localisations) and tracked by using the software HuygensPro (Scientific Volume Imaging). All PSFs with diameter larger than 20 pixels (1 µm/50.43 nm/pixel ≈ 20 pixels) were discarded to keep only the beads in focus in each acquired plane. Once all the selected beads were tracked, the software calculated the average velocity in the flow direction (V_x_) in each acquired plane position and the corresponding standard deviation.

### Calculation of flow velocity and shear stress fields

Flow tracing beads were identified and localised using the above mentioned ImageJ plugin (fig. s2b) using a sharpness box size of 40 × 40 pixels to minimise overlapping boxes due to the high density of beads in the perfusion medium. For all experiments, bead clusters, overlapping sharpness boxes and PSFs not fully contained in the sharpness boxes were discarded.

Flow tracks were created based on the CoM algorithm (fig. s2c). The analysis of the tracks has shown that some axial localisations were significantly (some microns) deviating from the main tracks, where the majority of the other points were located. These deviations are expected due to the different imaging conditions between calibration sample and immersion medium.

To reduce the impact of these inaccurate localisations, these were filtered out by using a code written in Mathematica (Wolfram). This code first discarded all points whose axial positions were outside the axial range and then removed all points whose axial positions were more than 1 µm different from those of the surrounding points. To remove these outliers (around 20% of the localisations), the second step assumed that the beads should not vary their relative axial positions likely more than 1 µm (i.e., around three times the average calculated standard deviation associated to the main tracks) over the exposure time period (100 ms) in laminar flow regime and in absence of obstacles as cells. After this filtering, the instantaneous velocities were obtained by calculating the gradient of the position changes along x, y and z directions over time via Excel (Microsoft).

For the validation experiment, the number of velocity data points (~ 1300 points) was reduced to improve the comparison with the velocity profiles calculated for the confocal microscope by firstly calculating the average axial position and velocity of every track, using Mathematica, then by averaging all the velocities associated to the points in a range of ± 0.50 μm from each integer axial position and positioning these average velocities on the central integer positions of the ranges. This averaging procedure resulted in one point (average velocity and standard deviation) per integer axial position (i.e. 9 points), as is the case for the profiles obtained with the confocal microscope. The velocity profiles obtained in the validation test were compared to those expected from the theory via eq. s6. For the experiments with cells, the filtering procedure described was then followed by the calculation of the associated velocity vectors. For the fixed cell experiment, however, the second step of the outlier filtering was updated to allow axial position variations up to 2 μm among adjacent localisations in the same track, due to presence of the cell perturbing the flow.

Homogeneous velocity maps have been generated through a MATLAB (MathWorks) code that was fitting all the unevenly distributed velocity components into the nodes of 3D regular grid (see supplementary material). Then, by applying the differentiations in eqs. s8, s9 and s10, the shear stress fields among the different x–y, x–z and y–z planes (i.e. S_xy_, S_xz_ and S_yz_) were calculated. All velocity and vector fields around the cell were 3D rendered by using the software Avizo (Thermo Fisher Scientific). In Avizo the velocity component fields were filtered by using a moving 3D Gaussian filter to reduce the fluctuations amplified by the calculations of the velocities. In addition, the velocities calculated at the nodes of the regular grid with the MATLAB code were linearly interpolated in Avizo to have continuous velocity maps. The shear stress maps were calculated in Avizo by using the velocity component fields and the shear stress equations, followed by filtering with the same Gaussian filter and interpolation used for the velocity components (fig. s2c).

### Cell culture and fluorescence staining

HeLa cells (ATCC, CCL-2) were used for the fixed cell experiment. Approximately 36,000 cells were seeded onto the PPFC and incubated at 37 °C to establish attachment. The cells were then fixed with 4% paraformaldehyde solution at room temperature for 20 min. Then, the fixing solution was washed with DPBS. MC3T3-E1 cells (Sigma-Aldrich, 99072810) and bone marrow derived hMSC (Lonza, PT-2501) were used in the live cell experiment. Approximately 50,000 MC3T3-E1 cells or hMSC were seeded onto the PPFC. These cells were then labelled with cell membrane stain DiD (Thermofisher Scientific, V22887) at 1:200 dilution in DMEM at 37 °C for 30 min, followed by washing with DPBS. Afterwards, cells were maintained in DMEM. Before imaging, DMEM-based perfusion media was added to the PPFC; cells were rested in the on-stage incubator at 37 °C for 30 min. To track cell morphology change over time during perfusion, a fluorescence micrograph excited with a 642 nm laser was captured on camera 1 every 15 min during perfusion, whereas the movement of the beads was recorded on camera 2 (Fig. 1a) of the MUM system. In addition, light transmission images of the cells were taken to confirm the fluorescence micrographs and used for visualisation purpose. The acquired fluorescence images were processed in ImageJ. The cells were identified through thresholding with Huang’s method^23^ of which the cell area and aspect ratio were measured.

## Results and discussion

### Validation of MUM for µPIV

**Figure 2:**
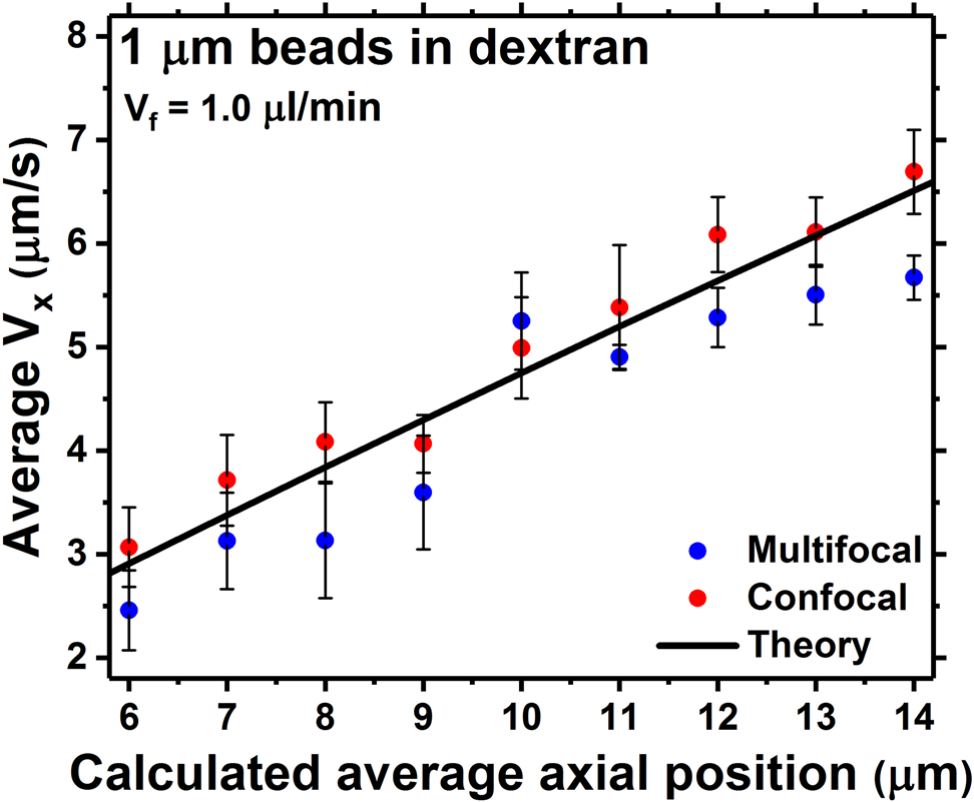
Theoretical V_x_ against axial position curve (black curve), compared to the experimental curves calculated from the bead flows acquired by using the confocal (red curve) and the multifocal (blue curve) microscopes. Vf = 1.0 µl/min.

The results of the validation study are shown in Fig. 2, where the velocity profiles calculated with the MUM system were compared to those from the gold-standard confocal microscope method alongside the theoretical curves (eq. s6). As mentioned, the curves in Fig. 2 have been acquired with V_f_ = 1.0 µl/min. In general, the curves from the confocal and the multifocal approaches tend to follow the theoretical curves, with the former slightly overestimating the theoretical behaviour and the latter slightly underestimating it.

The accuracies on the calculated velocities, estimated as the absolute values of the differences between calculated and theoretical values, on average are equal to (0.52 ± 0.19) µm/s and to (0.23 ± 0.11) µm/s for the multifocal and the confocal microscope. Regarding the precisions on the velocities (the error bars in Fig. 2), on average these are equal to (0.37 ± 0.14) µm/s and to (0.41 ± 0.09) µm/s for the multifocal and the confocal microscope. Hence, in the studied axial range, MUM had a slightly reduced axial accuracy but with comparable precision to confocal microscopy. The MUM reduced accuracy in this proof of concept work is most likely linked to factors including difference in refractive indices of agarose calibration sample and dextran solution (see supplementary material), depth-dependent spherical aberration^24,25^, aberrations arising from imaging at non-design plane for the used objective lens^26^, the impact of background light and reduced signal to noise ratio caused by splitting the signal among the acquired planes and the acquisition of defocused image^14^. Additionally, the little differences between experimental and theoretical curves in Fig. 2 cannot be imputed to the Brownian diffusion of the beads. Indeed, using the relations in Murphy^16^ and the number of localisations mentioned above, the Einstein diffusion coefficient is around 8.5 × 10^–16^ m^2^/s (at T ≈ 17 °C) and, hence, the worst case relative error on velocity due to Brownian motion is in the order of 0.1% (i.e., 0.003 μm/s at V_x_ = 3 µm/s). For the relative accuracy on the set flow rate, this also cannot be held responsible for the small differences in Fig. 2 between theory and measurements. Indeed, this is reported as ± 0.5% in the manual for the syringe pump, which turns into an absolute accuracy of ± 5 × 10^–3^ μl/min on a 1 μl/min flow rate. This roughly implies ± 0.025 μm/s with respect to a central measured velocity profile of V_x_ ≈ 5 μm/s (Fig. 2), i.e., around twenty times smaller than the calculated average accuracy for MUM.

If compared to other results in the literature^27,28^, the achieved accuracy is comparable whilst offering significant improvements in imaging speed. Each confocal image contains 1024 × 512 points and has been acquired in 261 ms. For a direct comparison, if the number of points is reduced to the number of pixels in each multifocal channel (~ 512 × 170 pixels), the same frame could be acquired in around 44 ms. However, this is the time to acquire a single plane. To cover the 8 µm axial range captured by MUM in 100 ms, i.e., 81 planes over the calibration phase, a laser scanning confocal microscope will take more than 3.5 s. If, instead, a spinning disk confocal microscope had been used (despite drawbacks as the increase in out of focus light and the less uniform field of illumination^29,30^), this would have taken around 1 s, assuming the camera was run at its maximum speed using 1/3rd of the full chip size (~ 77 fps). Consequently, in comparison to confocal microscopy, MUM offers a huge advantage to µPIV in terms of temporal resolution. Obviously, confocal microscopy still brings the strong advantage of efficiently discarding out of focus background light, thus raising the signal to background ratio and improving the localisation accuracy^29^. This, despite the loss in 4D imaging capabilities, in the context of μPIV can be a useful characteristic, especially in conditions of high emitter densities. However, provided the photon flux is adequate and the emitters can be properly distinguished, in MUM the out of focus light is efficiently exploited via its multiplane system to accurately localise the emitters over extended volumes (fig. s1), thus taking to instantaneous 4D imaging^12–15^.

From the validation study in Fig. 2 it can be concluded that MUM is a viable method to perform µPIV. MUM offers the important and clear advantage of fast 3D volume imaging, thanks to the simultaneous multifocal acquisition. This is very convenient with dynamic systems (e.g. cells adapting their morphology to the shear stress), where it might be needed to quickly track the variations in the behaviour of the flow to, in turn, follow the stresses applied by the latter and connect them to the responses of the systems under study.

### Reconstruction of velocity and shear stress maps around a fixed HeLa cell

**Figure 3:**
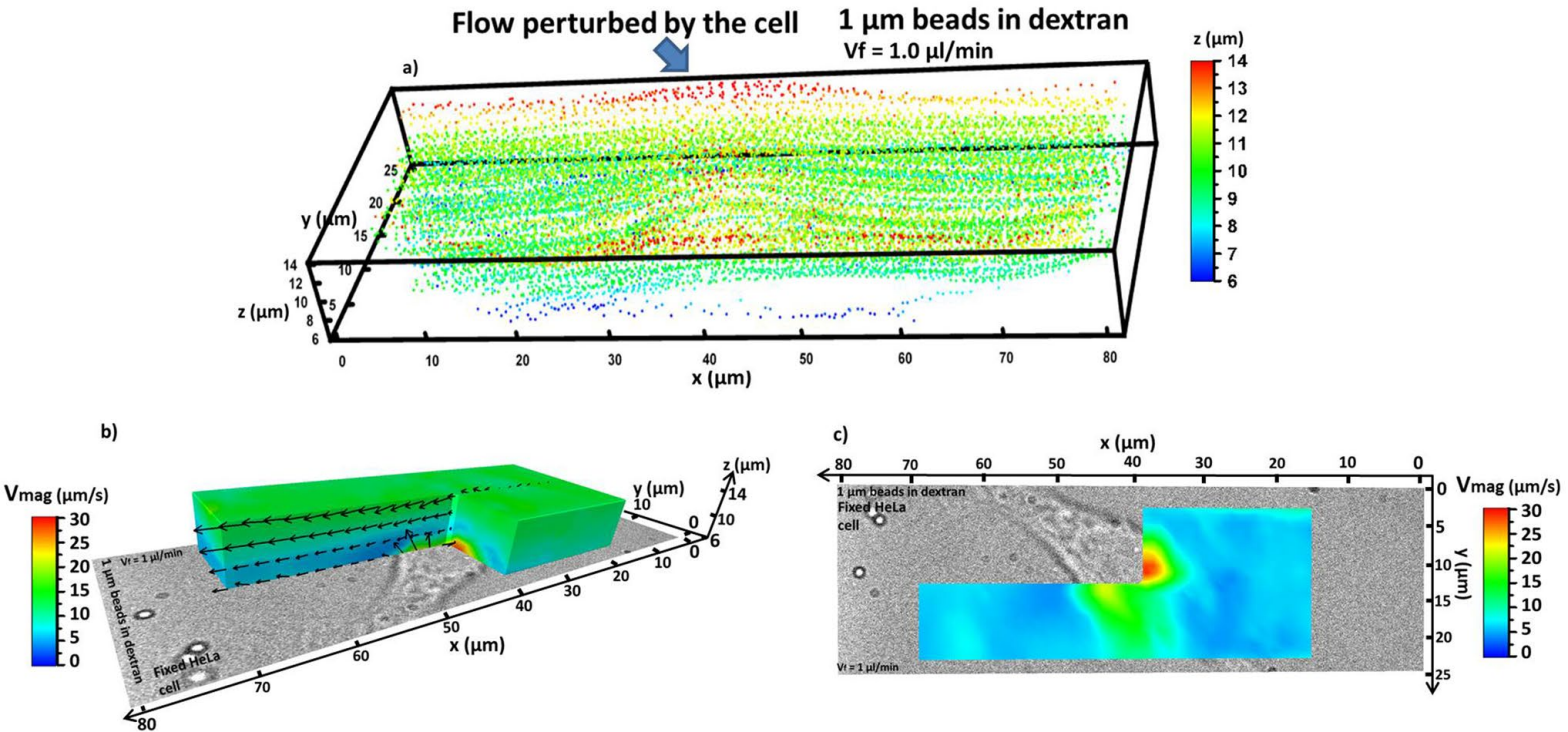
(**a**) 3D plot showing all localised 1 µm beads in dextran flowing over the selected HeLa cell. The perturbation to the flow generated by the cell is indicated. The axial positions are colour encoded in the plot. (**b**) Side view of the 3D map of the velocity magnitude associated to the fluid of 1 µm beads in dextran flowing around the fixed HeLa cell. The fluid flows from right to left along the x axis. (**c**) Bottom view of the velocity map in (**b**).

All localisations obtained from the fixed cell experiment are shown in Fig. 3a, with the axial positions colour encoded (over 22,000 localisations in 17,000 frames, grouped into more than 500 tracks). As can be seen, the flow appears perturbed around the centre of the plot, where the cell is positioned. As the beads flow over the cell, their axial positions tend to increase and subsequently decrease.

The total velocity magnitude (V_mag_) obtained by following the procedure described in the supplementary material and by using eq. s7 is presented in Fig. 3b (side view) and 3c (bottom view), together with selected vector planes. The individual velocity components V_x_, V_y_ and V_z_ are shown in the supplementary material (fig. s3). V_mag_ presents all the characteristics of the three velocity components, since it represents the intensity of the vector field and the overall behaviour of the flow. In those regions far from the cell position, V_mag_ behaves as a laminar flow. Near the cell position V_mag_ is maximised (28.6 µm/s) due to the presence of the velocity components perpendicular to the flow direction, i.e. V_y_ and V_z_, generated by the cell shape.

The perturbation to V_mag_ around the cell is curved, which follows the profile of 2D cultured adherent cells^31^. V_x_ velocities at positions far from those of the cell (fig. s3a,b) were lower than those of a laminar flow in absence of cells. In particular, at the z positions of 6 µm and 12.6 µm (i.e. the axial extremes of the field plots) V_x_ was equal to around 6.7 µm/s and 16 µm/s, while, following the theoretical equation (eq. s6), it should have been 11.3 µm/s and 22 µm/s. This was consistent with already established results that measured flow velocities became lower than the theoretical ones when perturbed by the presence of cells and tended to decrease as the cell density grows^4^.

**Figure 4:**
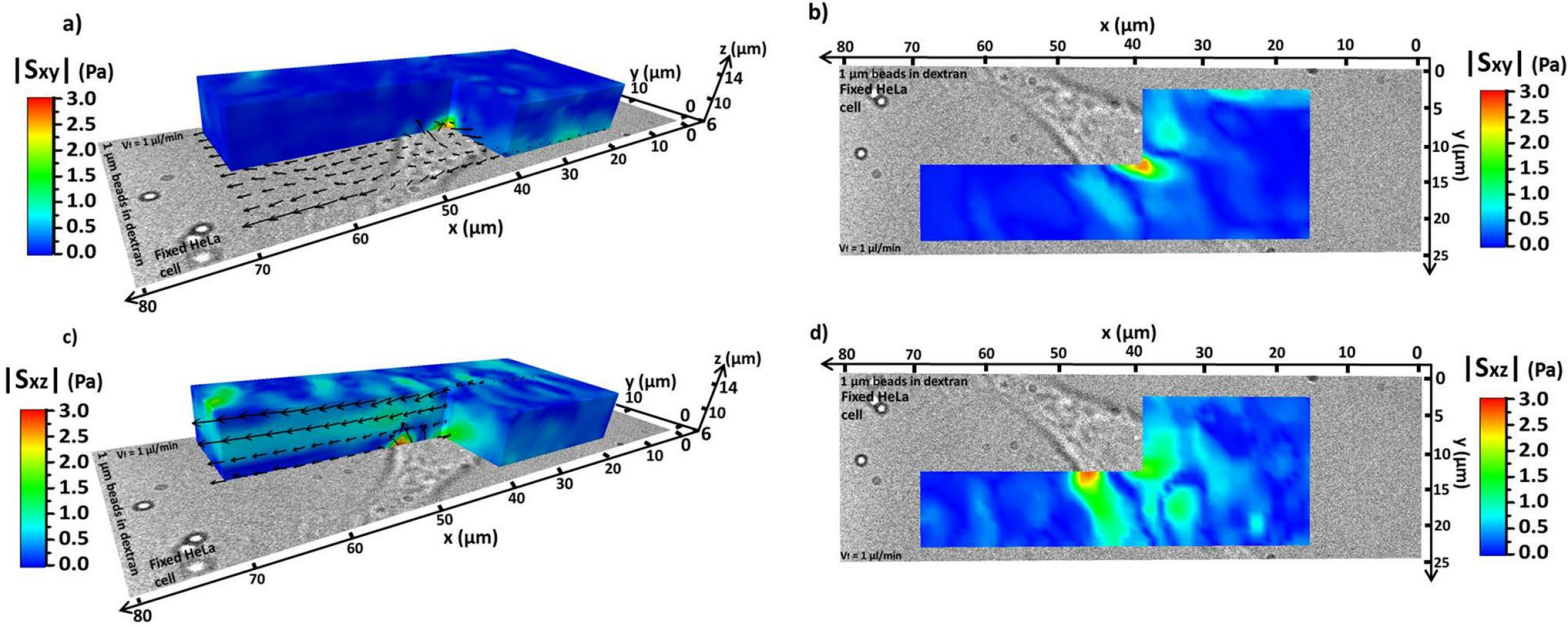
3D maps of the absolute values of the shear stress among different planes generated by the fluid of 1 µm beads in dextran flowing around the used fixed HeLa cell. The fluid flows from right to left along the x axis. The images in (**a**) and (**c**) show the side view, while those in (**b**) and (**d**) the bottom view. S_xy_ is shown in (**a**) and (**b**) S_xz_ in (**c**) and (**d**).

Figure 4 shows the magnitudes of the shear stress fields S_xy_ and S_xz_ around the cell (the S_yz_ field is shown in fig. s3 for completeness). As expected, in all the maps the shear stress results were maximised around the cell. Starting from S_xy_, i.e. the shear stress among x–y planes (Fig. 4a,b, eq. s7), its magnitude was maximised (≈ 2.5 Pa) in the same region V_mag_ had its peak (Fig. 3c). The same effect is seen looking at S_xz_, i.e. the shear stress among x–z planes (Fig. 4c,d).

The vectors in Fig. 4a,c, also showed a strong deviation toward the respective y and z directions in the regions where S_xz_ increases. In particular, S_xz_ reached a value of around 1.7 Pa. Another aspect to notice in Fig. 4c was the presence of a local high S_xz_ band (≈ 0.7 Pa), localised around the middle of the observed axial range. Some simulations in the literature^32^ showed that obstacles in the flow can introduce similar high velocity and shear stress bands. The band observed in Fig. 4c was generated by the shear between the fluid layers in the x–z planes due to the fluid first climbing up and then down the cell, as deducible from the vector field behaviour. This shear stress middle band was not present in the S_xy_ plot (Fig. 4a), while it was observable only in the shear fields that had an axial velocity V_z_ component (Fig. 4c and fig. s3g). Therefore, V_z_ must be directly responsible. In all the 3D shear stress fields in Fig. 4 it is clearly visible how the shear stress increased around the cell position. Far from the cell, some local maxima were also observed in the S_xy_, S_xz_ and S_yz_ plots, which may be caused by the slightly reduced accuracy of MUM.

In summary, MUM results on flow velocity and shear stress followed what would be expected for a cell perturbation in the middle of the flow and are consistent with previous works in the literature whilst offering considerable gains in 4D frame rates^4^.

### Change of cell morphology and local flow under perfusion in real time

**Figure 5:**
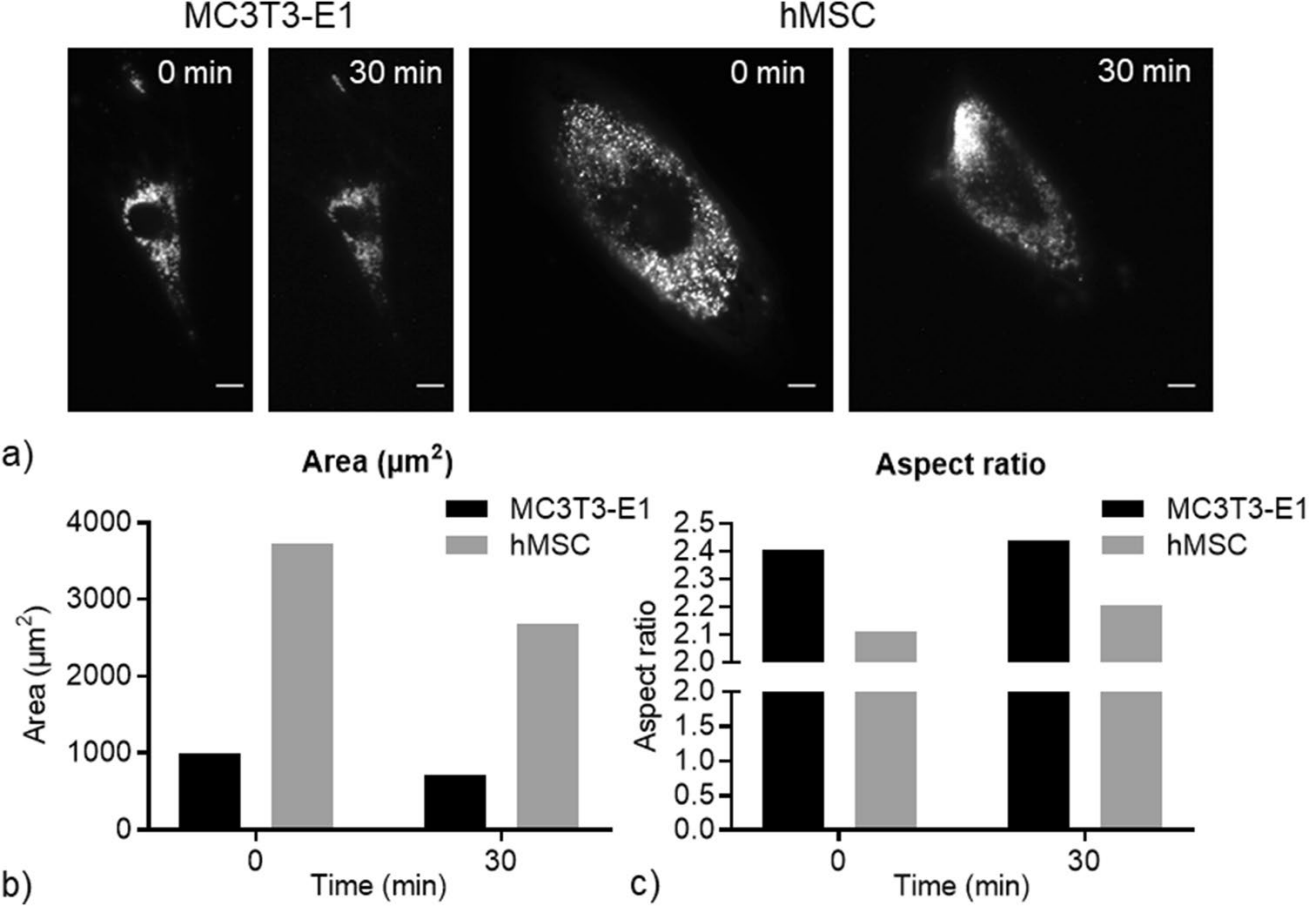
Cell morphology change during perfusion. (**a**) Fluorescent images of the MC3T3-E1 cell and hMSC before and after 30 min perfusion at 4 µl/min flow rate. Scale bar is 10 μm. (**b**,**c**) Quantification of cell morphology change including area and aspect ratio, respectively. Data shows a single cell as an exemplar.

Figure 5a shows the change of cell morphology of fluorescence labelled MC3T3-E1 and hMSC during 30 min perfusion. Quantitative data (Fig. 5b) revealed that the MC3T3-E1 cell was significantly smaller than hMSC, as expected from the literature^33,34^. The elongated morphology of the MC3T3-E1 and hMSC (Fig. 5c) were also reported previously^35,36^. For both cell types, 30 min flow perfusion led to reduced cell area and increased aspect ratio. Likewise, Horikawa et al.^37^ saw a decrease in area and an increase in aspect ratio of MC3T3-E1 cells after 1 h perfusion with 1 Pa WSS. Additionally, MC3T3-E1 cells were shown to be aligned with the flow direction after 6 h and 12 h. No significant cell orientation change was found in the current study, which could be caused by the relative short time under perfusion for cell movement.

**Figure 6:**
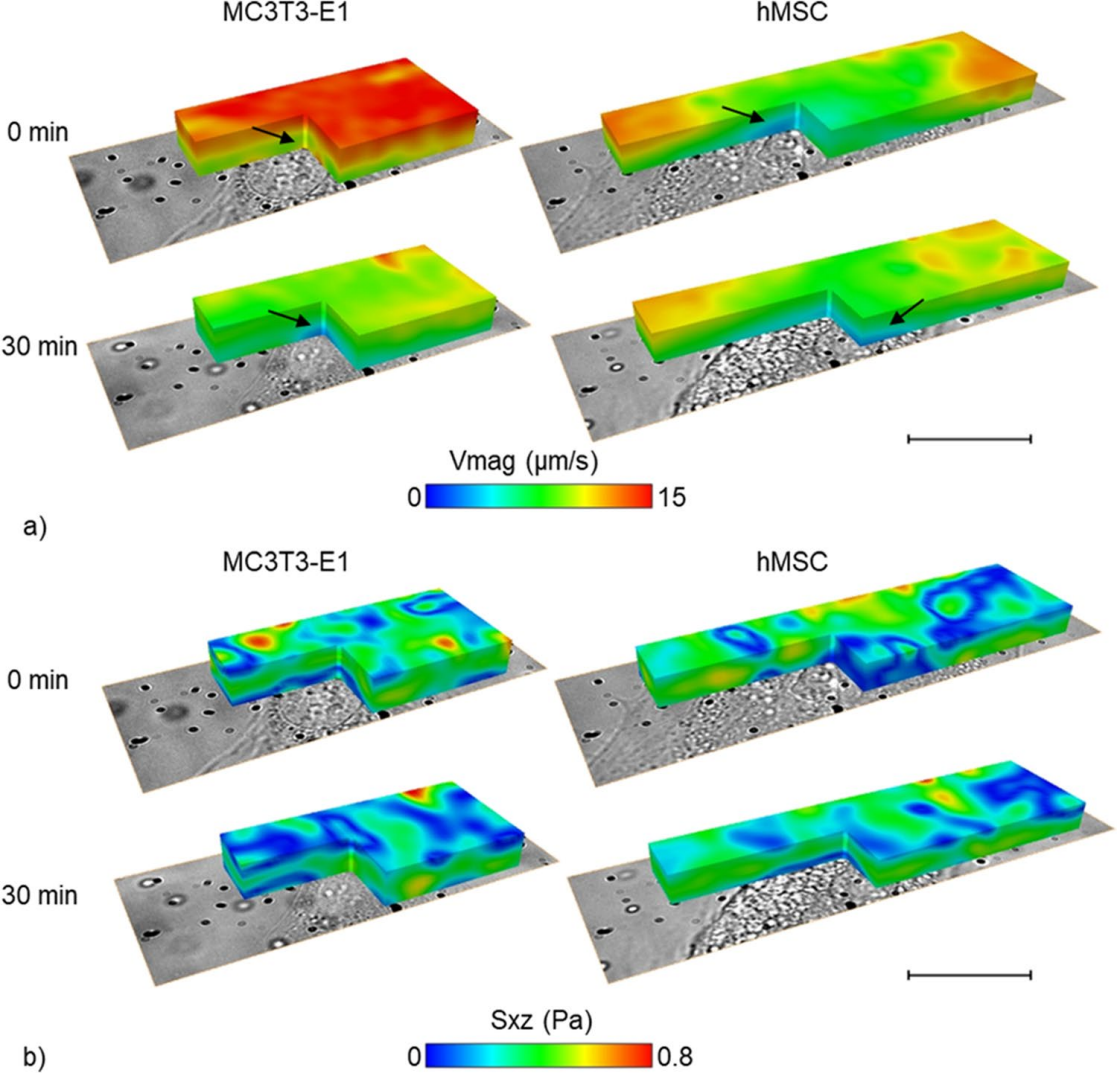
3D maps of the velocity magnitude (**a**) and absolute values of S_xz_ (**b**) around live MC3T3-E1 and hMSC cells before and after 30 min perfusion. Scale bar is 20 µm. Arrows indicate low velocity regions. The fluid flows from left to right.

For each live cell experiment, over 20,000 localisations, grouped into more than 1000 tracks were acquired. For both MC3T3-E1 and hMSC cell types, the velocity and FSS maps revealed the disturbance of the cells to the flow during the live cell study (Fig. 6). Unlike the fixed cell study, here the velocity component (V_x_) along the applied flow direction dominated the velocity magnitude whereas the perpendicular (V_y_) and axial (V_z_) velocity component did not have a significant impact (fig. s4). Lower V_y_ and V_z_ led to reduced FSS contribution from S_xy_ and S_yz_ components (fig. s5). Similar to the fixed cell study, increased FSS was observed at the peripheral of the cells.

At 0 min, on average, the velocity magnitude of the flow around the MC3T3-E1 cell was much higher than that around the hMSC (Fig. 6a). Inside a PPFC, higher velocity magnitude generally indicates longer distance to the channel wall^8^. Since comparable axial (z) position was used during the data acquisition, it showed that the MC3T3-E1 cell had a much lower cell height compared to the hMSC at 0 min. There is no direct comparison on cell height between MC3T3-E1 and hMSC in the literature. With atomic force microscope (AFM), Docheva et al.^38^ demonstrated that hMSC (~ 2 µm) had significantly lower cell height compared to osteosarcoma MG63 cells (~ 4.8 µm). Also using AFM, Andersen et al.^39^ measured the height of MC3T3-E1 cells on the tantalum substrate to be around 2.4 µm. The velocity magnitude around the MC3T3-E1 cell reduced greatly after 30 min perfusion, showing an increase in cell height. In comparison, the reduction in flow velocity in hMSC was less apparent at 30 min, which indicates a more gradual cell height change. It is worth noting that the region with low velocity magnitude shifted for hMSC cells, indicating a surface topography change of the cell. Previously, computational model results demonstrated that FSS could lead to cellular deformation^40^. The limited cell morphology change of hMSC could result from a higher cellular stiffness compared to MC3T3-E1 cells^41,42^.

From Fig. 6b, stress hotspots were seen at the top surface of both MC3T3-E1 and hMSC. Stress hotspots were also observed by Rossi et al.^43^, which located around the apex of a single chicken endothelial cell. For the MC3T3-E1 cell, S_xz_ was higher with more stress hotspots at 0 min than 30 min, which was caused by the elevated flow velocity. However, the average S_xz_ at the cross-section sides of the cells at 0 min and 30 min were comparable (≈ 0.4 Pa). The S_xz_ magnitude around the hMSC did not change significantly during perfusion. Nevertheless, it can be seen that the stress hotspot relocated according to the regions with low flow velocity discussed above, again indicating the influence of cell topography on the local flow. Furthermore, for both cell types, the average FSS experienced by the cell (≈ 0.4 Pa) was significantly lower than the applied WSS (0.86 Pa), which was previously reported by Song and colleagues^4^. They also found that cell seeding density significantly affects FSS around cells and observed that lower cell density led to greater FSS difference across cell thickness. Taken together, using MC3T3-E1 and hMSC cells as an exemplar, the FSS at cell edge varies from the applied WSS and is dependent on cell seeding density, which highlights the importance in quantifying the local FSS for mechanotransduction studies in order to obtain more consistent findings across studies. MUM was able to capture the whole volume of interest simultaneously, which offers significant advantage over confocal system that acquires data sequentially. Indeed, if the same confocal system used for the validation test had been used, the data acquisition phase would have taken ~ 1 h 40 min (see supplementary material) and all localizations would have been located at the acquired planes, thus considerably loosing axial resolution, together with the loss in 4D imaging. Morphology changes of MC3T3-E1 and hMSC cells used in this study were observed within 30 min of perfusion; fast acquisition speed (~ 300 times higher than laser scanning confocal, see supplementary material) of MUM enables researchers to study fast evolving biological systems.

## Conclusions

This paper has studied the application of MUM as a tool to perform µPIV to reconstruct the velocity and shear stress maps generated by the flow of a perfusion medium around a cell inside a PPFC. To this purpose, MUM was first validated against the theoretical expected behaviour and confocal microscopy. It showed that MUM achieved a good estimation of the axial velocity compared to the theoretical values. Compared to confocal laser scanning microscope, MUM had a slightly reduced accuracy with comparable precision but at a significantly faster acquisition speed.

After the validation experiment, MUM was used to reconstruct the velocity and shear stress profiles around a fixed HeLa cell, which clearly showed the perturbation to the flow caused by the cell. In general, the behaviour of the velocity and shear stress fields around the cell were comparable with what has been observed in other studies^32,44^. However, it was also important to notice the presence of some fluctuations in the calculated shear stress fields.

MUM was then used to assess the local flow around two cells with different morphology in real-time. It showed it was able to capture the local flow change caused by cell morphology difference and the evolution with time under perfusion. Also, it highlighted the importance of quantifying the local flow, which can be significantly different from the theoretical estimation when using a PPFC for mechanotransduction studies.

In this proof of concept work, we have demonstrated a 300 times improvement in acquisition speed using MUM over traditional confocal laser scanning microscopy whilst returning only slightly reduced accuracy and similar precision. This can be further optimized for specific imaging conditions through customization of diffraction grating and optical relay. This is a very important characteristic in situations where the biological systems under study evolve quickly and it is necessary to monitor in real time entire volumes to correlate the sample responses to the forces generated by the flow. Furthermore, the flow acquisition time could be further reduced, e.g., by increasing the bead density and reducing the camera exposure time, provided the ability to discriminate among emitters and have an adequate signal level are not compromised. Consequently, MUM can help to clarify some of the mechanisms through which organisms develop, grow and adapt while undergoing fluid shear stress.

## Supplementary Information

Supplementary Information.

## Data Availability

The datasets used and/or analysed during the current study are available from the corresponding author on reasonable request.
