# Supplementary material for: Instantaneous 4D micro-particle image velocimetry (µPIV) via multifocal microscopy (MUM)
Source: Sci Rep. 2022 Nov 2;12:18458. doi: 10.1038/s41598-022-22701-3 (PMC9630545; doi:10.1038/s41598-022-22701-3)
Supplement: Supplementary file 1 — Supplementary Information. [file 41598_2022_22701_MOESM1_ESM.docx]

Instantaneous 4D micro-particle image velocimetry (µPIV) via multifocal microscopy (MUM)

M. G. R. Guastamacchia^1,2^, R. Xue^3,4^, K. Madi^3,6^, W. T. E. Pitkeathly^5^, P. D. Lee^3,4^, S. E. D. Webb^2,7^, S. H. Cartmell^3,4^ and P. A. Dalgarno^6,*^

^1^ EPSRC Centre for Doctoral Training in Applied Photonics, Heriot-Watt University, Edinburgh, UK

^2^ Science and Technology Facilities Council, Research Complex at Harwell, Rutherford Appleton Laboratory, Harwell, UK

^3^ Department of Materials, School of Natural Sciences, Faculty of Science and Engineering, University of Manchester, Manchester, UK

^4^ The Henry Royce Institute, Royce Hub Building, The University of Manchester, Manchester, UK

^5^ Institute of Biological Chemistry, Biophysics and Bioengineering, Heriot-Watt University, Edinburgh, UK

^6^ Present address: 3Dmagination Ltd, Atlas Building, Harwell Campus, Didcot, UK

^7^ Present address: Biotechnology and Biological Sciences Research Council, Swindon, UK

*Corresponding author (PAD)

e-mail: p.a.dalgarno@hw.ac.uk

Supplementary material

# Sample preparation

Dextran, a polysaccharide ^1^, has been used to raise the viscosity and, consequently, the shear stress ^2^ imposed by the perfusion medium used for the validation and fix cell experiments. The density (ρ) and the refractive index (n) of this perfusion medium were calculated by using two empirical equations described in ^1^. These indicate that ρ (at room temperature and 1 atm) is equal to (eq. s1):

$\rho= 0.99717 \frac{g}{\mathrm{ml}} + 0.00398133 \frac{g}{\mathrm{ml}} d+ 0.00001597 \frac{g}{\mathrm{ml}} d^{2}$ (eq. s1)

and n (at a wavelength of 589.29 nm, 1 atm and room temperature) to (eq. s2):

$n= 1.33299+0.00151005 d+0.000006372 d^{2}$, (eq. s2)

where d is the number of grams of dextran per 100 g of solution. In this case d = 22.64 (assuming the density of the DPBS and the bead stock solution were equal to that of water, i.e. 1 g/ml) and, consequently, ρ = 1.10 g/ml (eq. s1) and n = 1.37 (eq. s2).

The viscosity of the perfusion medium has been measured by using a rheometer (TA Instruments, HR-3) and resulted to be 0.50 Pa∙s for the validation and fixed cell tests and 0.43 Pa∙s for the live cell experiment.

The wall shear stress (WSS) inside a parallel-plate flow chamber (PPFC) was estimated with eq. s3 ^3^ to be 1.00 Pa for the validation and fixed cell experiment and 0.86 Pa for the live cell experiment:

$\tau=\frac{6Q\mu}{wh^{2}}$, (eq. s3)

where τ is the WSS (Pa) of PPFC, Q is the flow rate (m^3^/s), µ is the viscosity (Pa∙s), w is the channel width (m) and h is the height of channel height (m).

**Particle localisation and tracking via MUM system**

The sharpness is a single metric that defines the level of optical aberration in an image. It minimizes at low aberration and, consequently, maximised at minimum aberration ^4^. On optimised imaging systems it is dominated by defocus as the primary aberration (fig. s1a). The algorithm employed in this work exploits a set of sharpness calibration curves (one per plane) from reference samples to determine the estimated defocus position of a particle in a single multifocal image. By comparing the sharpness values of a set of images to the corresponding sharpness curves via Gaussian probability density functions (PDFs), the most likely axial position can be estimated ^4^. The sharpness (S) of a 2D PSF can be calculated by using the following formula (eq. s4):

$\mathbf{S=}\frac{\sum_{\mathbf{k=1}}^{\mathbf{q}} \left( {\mathbf{n}_{\mathbf{k}}}^{\mathbf{2}}\mathbf{-}\mathbf{n}_{\mathbf{k}} \right)}{\left( \sum_{\mathbf{k=1}}^{\mathbf{q}} \mathbf{n}_{\mathbf{k}} \right)^{\mathbf{2}}}$, (eq.s4)

where n_k_ is the image count of the k^th^ pixel within a box (the sharpness box) of q pixels that contains a 2D PSF.

To produce the calibration sample, a support material as agarose gel with refractive index close to that of the used perfusion medium (n_agarose_ = 1.33) has been selected. The agarose calibration sample has been prepared by adding 40 mg of agarose (Sigma-Aldrich, A2576) and 30 μl of 1 μm yellow/green beads stock solution (Thermo Fisher Scientific, F13081) to 2 ml of distilled water. This prepared solution has been, first, manually stirred and put into a microwave for 30 s at maximum power to melt the agarose. Then, it has been poured into a glass-bottomed dish (MatTek Corporation, P35G-0.170-14-C), with the top side of the coverslip manually pre-pen marked to have a reference for the surface position. Finally, the agarose sample has been left into a fridge at 4˚C for at least 15 min - 20 min to let it cool down and solidify before imaging.

To calculate the sharpness curves and best correspond to the imaging conditions of typical cells on a coverslip, a bead positioned within the first 10 µm from the bottom of the glass-bottomed dish has been chosen. The following imaging parameters have been used for the calibration sample: exposure time (ET) = 100 ms, electron multiplying (em) gain = 2,000 and laser power at sample = 87.18 μW at λ = 488 nm. A series of ten z-stacks has been acquired to build the sharpness curves by, first, focusing the selected bead on the central plane and, then, by moving the objective lens, with a nanometric precise piezo (Physik Instrumente, E-625 and P-721 PIFOC), by 100 nm steps over the range of ±7 μm with respect to it.

In fig. s1a, a lateral cross section acquired with the MUM system and showing out of focus images of the selected bead in the three imaged planes is presented, together with the sharpness boxes within which the sharpness has been calculated (150 × 150 pixels). In fig. s1b the obtained sharpness curves are presented. In fig. s1c the calculated against nominal axial position curve, evaluated for the calibration bead in fig. s1a and by using the sharpness algorithm is shown. The error bars are the standard deviations calculated at every axial position. The axial range over which the calculated positions correspond to the nominal ones with an accuracy of at least 100 nm is equal to (8.00 ± 0.23) µm. Fig. s1d shows the standard deviations, i.e. the error bars in fig. s1c, plotted against the nominal axial positions. The average axial precision within the axial range is equal to (48.7 ± 13.2) nm. The lateral precision (fig. s1e) was obtained through centre of mass (CoM) calculations, which were performed on the z-stacks resulting from the sub-pixel precise lateral registration of the images in the three acquired planes. This is done by using pre-acquired z-projected z-stacks (one per plane), where multiple bright emitters are present. The alignment feature of the plugin, first, processes these images by using a Laplacian of Gaussian (LoG) filter ^5^ to reduce the noise and enhance the spot features and applies a threshold to detect bright objects. Secondly, it segments the resulting images to identify the spots and discard those made by a number of pixels below a certain threshold. Finally, the spots that have passed the segmentation step are 2D localised in each z-projection by calculating their centres of mass (CoMs). The 2D localisations associated to the non-zeroth orders are then laterally translated by the plugin to be aligned to the corresponding ones on the zeroth order. The alignment procedure is performed by providing to the plugin a translation distance, which takes the corresponding spots on the different planes close to each other, and a pairing distance, which


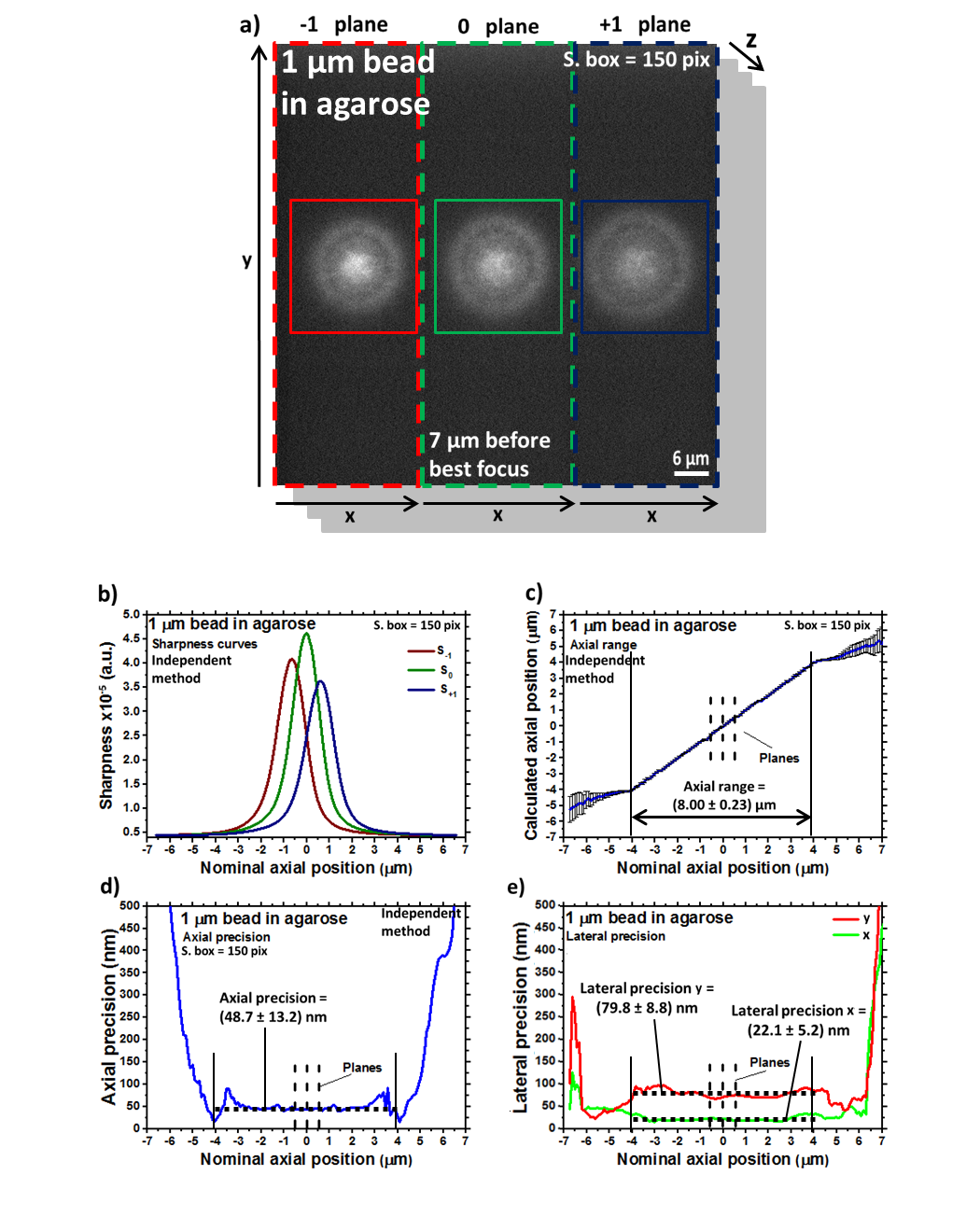


**Figure s1 a)** Axial cross section showing the 1 µm bead in agarose used to build the sharpness curves. **b)** Sharpness curves calculated to reconstruct the flow velocity profile. A sharpness box of 150 pixels has been used. **c)** Calculated axial position against nominal axial position curve. **d)** Axial precision against nominal axial position curve. **e)** Lateral precision over x and y directions against nominal axial position curves.

is used to finely translate the images and find the best overlap between the CoMs within it. The average x and y lateral precisions within the axial range are, respectively, (22.1 ± 5.2) nm and (79.8 ± 8.8) nm (the precision over the y direction is worse than that over the x one potentially due to some non-optimal calibration sample positioning on the sample holder). The plane registration function, the sharpness and the CoM calculations described have been performed by using an ImageJ (National Institutes of Health) plugin. The calibration curve calculation process is schematised in fig. s2a.


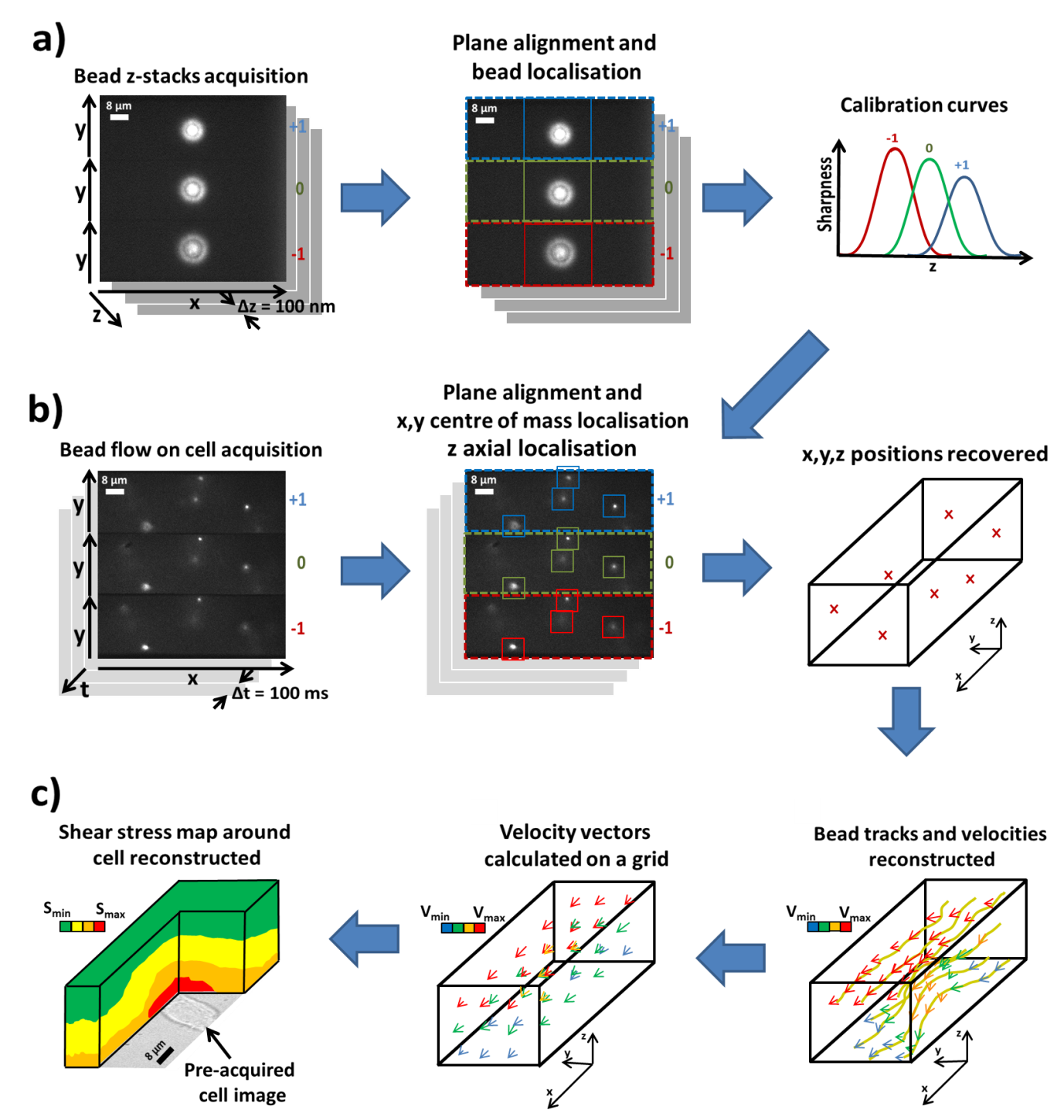


**Figure s2** Schematisation of the steps used to generate velocity and shear stress maps via the MUM setup showed in fig. 1a. The acquisition of a calibration sample **a)** allows to calculate the sharpness curves, which are then **b)** used to determine the axial positions of the beads flowing around a selected cell (whose transmission image has been pre-acquired). These axial positions are joined with the corresponding x-y ones determined via the used CoM algorithm and used to create tracks **c)**, which inform on the flow behaviour. The tracks are then used to obtain the desired velocity and shear stress maps.

The plane registration function, the sharpness and the CoM calculations described have been performed by using an ImageJ (National Institutes of Health) plugin. The calibration curve calculation process is schematised in fig. s2a.

Regarding the beads in the flows observed via the MUM relay, the same plane registration and CoM algorithms above described have been used. Concerning the axial localisation, instead, a sharpness box size of 40x40 pixels has been selected, to avoid the overlap among sharpness boxes due to the density of beads in the perfusion medium. To minimise the impact of the different levels of background included in the sharpness boxes, a modified version of the sharpness algorithm that involves ratiometric sharpness curves has been used. In the ratiometric algorithm the ratiometric sharpness curve in the j^th^ plane as a function of the axial position z of the plane (R_j_(z)) can be calculated directly from the sharpness curves in each i^th^/j^th^ plane (S_i/j_(z)) in fig. s1b as eq. s5:

$R_{j}\left( z \right)= \frac{S_{j}\left( z \right) - S_{\mathrm{Bj}}}{\sum_{i= -m}^{+m} \left( S_{i}- S_{\mathrm{Bi}} \right)}$, (eq. s5)

where S_Bi/j_ is the sharpness associated to the background in each plane and can be assumed to be equal to the smallest sharpness value of each sharpness curve.

Since the sharpness associated to the background is pre-subtracted from the sharpness curves, the impact of the background on the sharpness values is reduced. In the sharpness algorithm the PDFs are slightly modified, since the variance of the sharpness curves is assumed as equal to the ratiometric curves squared. The process to localise the beads in the bead flow acquired via the MUM relay is shown schematically in fig. s2b.

After having manually discarded all localisations of bead clusters (recognised by their irregular non-circularly symmetric shapes and intensity profiles) and where the sharpness boxes were overlapping or not fully containing the PSFs, all beads localised in the flows acquired via the MUM setup have then been joined into tracks. This has been done by using the tracking function inserted in the above mentioned MUM plugin, which exploits a mixed Kalman filter ^5,6^.

Concerning the bead density observable in fig. s2b and 1c, this is consistent with the number of acquired frames and localisations. Indeed, assuming for the fixed cell experiment a total number of localisations of 27,500 (including the 20% discarded ones) and 17,000 frames, the average number of acquired localisations per frame is around 1.6, i.e., in the same order of magnitude of the number of beads visible in fig. 1c and s2b.

# Calculation of flow velocity and shear stress fields

A code written in Mathematica (Wolfram) was used to remove particle localisation outliers by firstly discarding all points whose axial positions were outside the axial range, and then discarding all points whose axial positions were more than 1 µm different from those of the surrounding points. The first step has been performed to remove all those points localised before or after the axial range boundary, since these are not considered accurate. The second step is based on the assumption that the beads are not expected to massively vary their axial positions over the exposure time period.

The V_x_(z) profiles obtained in the validation test have been compared to those expected from the theory via eq s6 ^2^:

$V_{x}\left( z \right)= \frac{6 V_{f}}{h^{3}w}(hz-z^{2})$, (eq. s6)

which is valid far from the perturbations to the flow and for a mono-dimensional flow along the flow direction x.

The MATLAB code created a grid covering the fields of view (FOVs) acquired with the MUM (26x81x8 µm) and has the nodes spaced 1 µm along x, y and z directions. By combining the homogeneous V_x_, V_y_ and V_z_ fields obtained in this way, it is possible to achieve a 3D map of the velocity magnitude (V_mag_, eq. s7) and vectors evenly distributed around the cell (fig. s2c):

$V_{\mathrm{mag}}\left( x,y,z \right)= \sqrt{{V_{x}}^{2}\left( x,y,z \right)+{V_{y}}^{2}\left( x,y,z \right)+{V_{z}}^{2}\left( x,y,z \right)}$. (eq. s7)

Then, by applying the differentiations in eq. s8, s9 and s10^7^, the shear stress fields among the different x-y, x-z and y-z planes (i.e. S_xy_, S_xz_ and S_yz_) can be calculated:

$S_{\mathrm{xy}}\left( x,y,z \right)= \mu\left( \frac{\partial V_{x}(x,y,z)}{\partial y}+\frac{\partial V_{y}(x,y,z)}{\partial x} \right)$, (eq. s8)

$S_{\mathrm{xz}}\left( x,y,z \right)= \mu\left( \frac{\partial V_{x}(x,y,z)}{\partial z}+\frac{\partial V_{z}(x,y,z)}{\partial x} \right)$ (eq. s9)

and $S_{\mathrm{yz}}\left( x,y,z \right)= \mu\left( \frac{\partial V_{y}(x,y,z)}{\partial z}+\frac{\partial V_{z}(x,y,z)}{\partial x} \right)$. (eq. s10)

# Reconstruction of velocity and shear stress field maps around the fixed HeLa cell

To have a more detailed view of the behaviour of the flow around the cell, the magnitude of the velocity components (V_x_, V_y_ and V_z_) and of the S_yz_ field are presented separately in fig. s3, together with some selected vector planes.


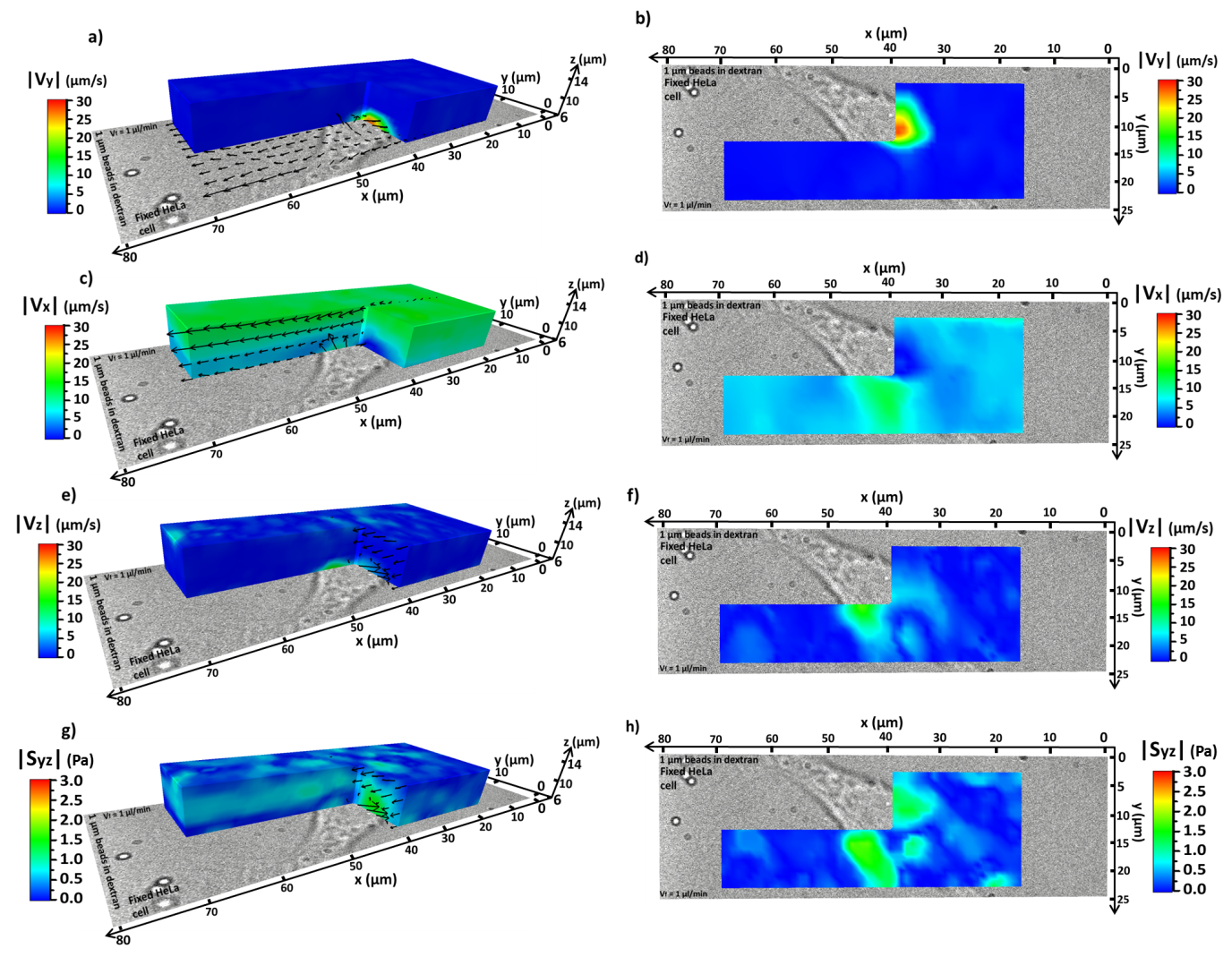


**Figure s3** 3D maps of the absolute values of the velocity components and of the S_yz_ shear stress maps associated to the fluid of 1 µm beads in dextran flowing around the chosen fixed HeLa cell. The fluid flows from right to left along the x axis. The images in **a)**, **c)**, **e)** and **g)** show the side views, while those in **b)**, **d)**, **f)** and h the bottom views. V_y_ is shown in **a)** and **b)**, V_x_ in **c)** and **d)**, V_z_ in **e)** and **f)** and S_yz_ in **g)** and **h)**.

# Change of cell morphology and local flow under perfusion in real time

The overall velocity magnitude around the MC3T3-E1 and hMSC during the live cell experiment was largely affected by the velocity component V_x_, which was along the flow direction (fig. s4a). In contrast, the magnitude of V_y_ and V_z_ components was significantly lower. Nevertheless, cell location could also be identified from the region with low magnitude of V_y_ in fig. s4b. Cell location was less apparent from V_z_ plots (fig. s4c) due to the topographical features on the cell surface. For the MC3T3-E1 cell at 30 min, a hotspot was observed, indicating a protrusion generated from the cell during morphology change under perfusion.


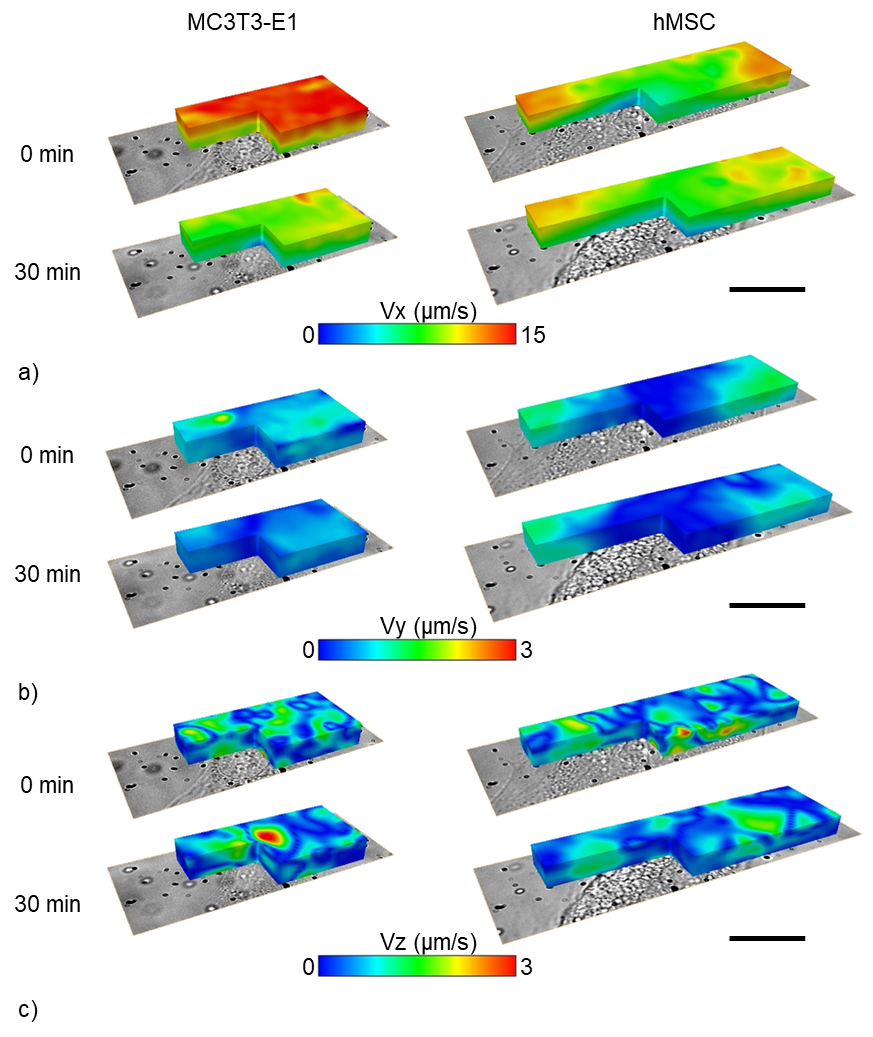


**Figure s4** 3D maps of absolute values of the velocity components around live MC3T3-E1 and hMSC cells before and after 30 min perfusion. Scale bar is 20 µm.

The shear stress along the x-z planes (S_xz_) has a bigger impact than S_xy_ and S_yz_ since V_x_ was the highest and its change along z axis was the most significant (fig. s5). Generally, increased shear stress magnitude was observed near the cells, and stress hotspots were likely caused by the topographical features of the cells.

For the live cell experiment the time potentially needed for the used confocal system to capture the same imaging volume covered by MUM was calculated. First, number of pixels in each MUM channel was calculated as 421 × 1,972 = 830,212 pixels. The confocal system used (Leica, TCS SP8) was able to capture 1,024 × 512 = 524,288 pixels in 261 ms at the maximum speed. Therefore, to capture the same number of pixels in a MUM channel, the laser scanning confocal system would need around 413 ms (830,212/524,228 × 261 ms ≈ 413 ms). Since there are 81 frames in the calibration image sample in the 8 µm range covered by the MUM system, the used confocal system might roughly take 33.5 s (0.413 s × 81 ≈ 33.5 s) to capture the same volume, i.e., ~335x slower. Concerning the time specifically needed by the confocal system used for the validation test to obtain the same localisations achieved by MUM in 10 min, some rough calculations can be set down. Assuming 20,000 localisations per live test (i.e., MC3T3-E1 and hMSC cell tests at 0 min and 30 min) and that the confocal system achieved 5,700 localisations in 18 min with an exposure time of 261 ms, the same system would have realised ~3,600 localisations with an exposure time of 413 ms (i.e., 5,700 × 0.261 s/0.413 s). Consequently, it would have taken ~1 h 40 min to the used confocal microscope to get 20,000 localisations (20,000 × 18 min/3,600). Additionally, all these localisations would have all been located over the 9 acquired planes spaced 1 μm, thus considerably loosing axial resolution, together with the loss in 4D imaging.


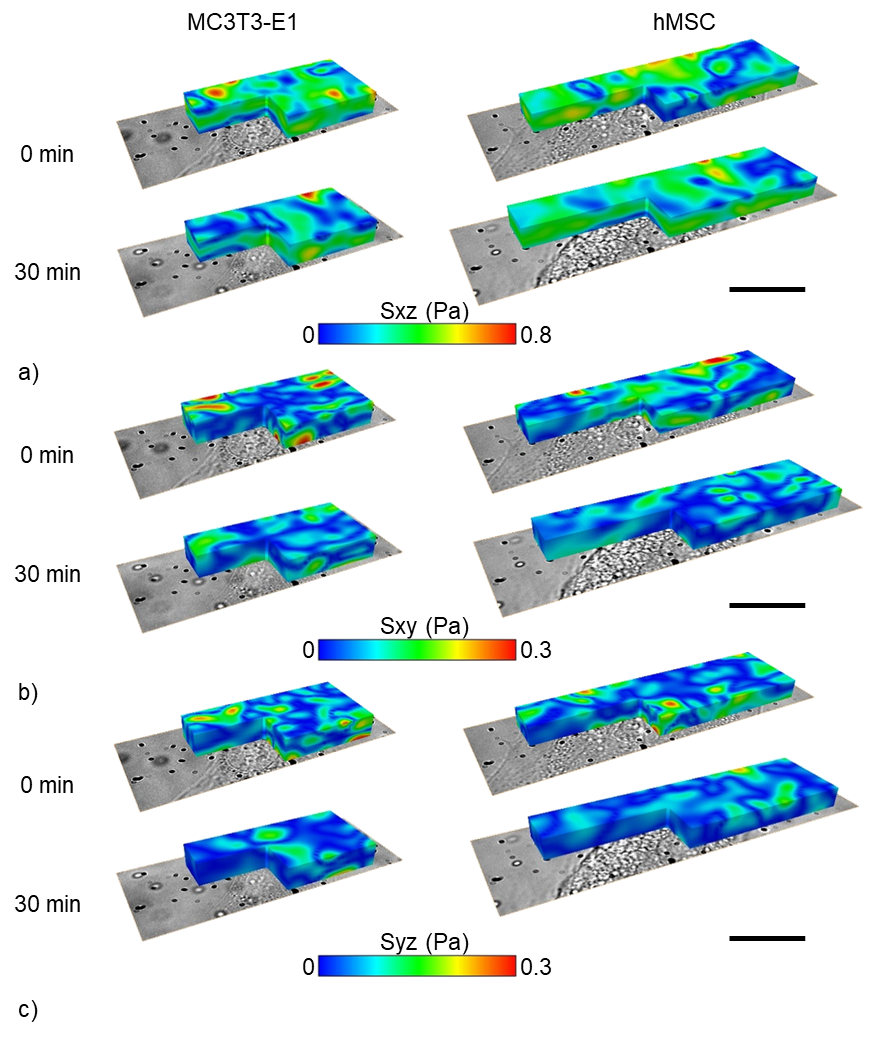


**Figure s5** 3D maps of absolute values of the shear stress components around live MC3T3-E1 and hMSC cells before and after 30 min perfusion. Scale bar is 20 µm.

# References

1 Snyder, C., Isbell, H., Dryden, M. & Holt, N. Optical rotations, refractive indices, and densities of dextran solutions. *Journal of Research of the National Bureau of Standards* **53**, 131-137 (1954).

2 Nakayama, Y. *Introduction to fluid mechanics*. (Butterworth-Heinemann, 2018).

3 Michael Delaine-Smith, R., Javaheri, B., Helen Edwards, J., Vazquez, M. & Rumney, R. M. Preclinical models for in vitro mechanical loading of bone-derived cells. *Bonekey Rep* **4**, 728, doi:10.1038/bonekey.2015.97 (2015).

4 Dalgarno, H. I. *et al.* Nanometric depth resolution from multi-focal images in microscopy. *J R Soc Interface* **8**, 942-951, doi:10.1098/rsif.2010.0508 (2011).

5 Kalman, R. E. A new approach to linear filtering and prediction problems. (1960).

6 Fukunaga, K. & Flick, T. E. An optimal global nearest neighbor metric. *IEEE transactions on Pattern analysis and Machine Intelligence*, 314-318 (1984).

7 Boucher, R. F. & Nakayama, Y. in *Introduction to Fluid Mechanics* 87-94 (Butterworth-Heinemann, 2000).
